# Serine biosynthetic pathways signal to diverse outputs in plants

**DOI:** 10.1093/plcell/koad272

**Published:** 2023-10-25

**Authors:** Lucas Frungillo

**Affiliations:** Assistant Features Editor, The Plant Cell, American Society of Plant Biologists; Institute of Molecular Plant Sciences, School of Biological Sciences, University of Edinburgh, Edinburgh, UK

Serine (Ser) is a central structural and signaling amino acid in cells. Among its various roles, Ser is a building block of proteins, an indispensable substrate for the biosynthesis of phospholipids and nucleotides, and is one of the only 3 amino acids phosphorylated by kinases in peptides (Kalhan and Hanson 2012). In plants, Ser is synthesized through 3 different pathways (reviewed by Ros et al. 2014). While the glycolytic intermediate 3-phosphoglycerate feeds both the glycerate pathway and the phosphorylated pathway of Ser biosynthesis (PPSB), photorespiration promotes the glycolate pathway of Ser biosynthesis (GPSB). Because of its importance to cellular homeostasis, Ser metabolism is tightly regulated, and the relevance of each biosynthetic pathway has been the focus of intense debate.

In this issue of *The Plant Cell*, **Sara Rosa-Téllez, Andrea Alcántara-Enguídanos, and colleagues (**Rosa-Téllez et al. 2023**)** show that manipulation of flux through the GPSB relays nutritional status in shoots and regulates the growth of PPSB mutant Arabidopsis plants. This work is a fine example of how selective bioengineering of intertwined cellular metabolic pathways has the potential to improve plant yield.

Recently, the authors revealed that PPSB-derived Ser promotes Arabidopsis growth by driving cell division and elongation. Consequently, PPSB mutants display reduced biomass accumulation compared to wild-type plants (Zimmermann et al. 2021). However, whether GPSB can be engineered to compensate for PPSB suppression remained unknown. In the GPSB, the conversion of glycolate into glycine (Gly) represents a branching point in Ser biosynthesis, because Gly is either converted into Ser by the activity of serine hydroxymethyltransferase (SHMT1) or deaminated in mitochondria by the Gly decarboxylase complex to generate NADH and 5,10-methylene-tetrahydrofolate. To avoid the pleiotropic effects of manipulation of photorespiration, Rosa-Téllez and colleagues cleverly engineered photorespiratory Ser biosynthesis by targeting the GPSB branching point. By mutating *SHMT1*, thus diverting Gly from Ser synthesis in the GPSB, the authors established a novel framework to dissect the cross-talk between different Ser biosynthetic pathways in cells. Phenotypic analysis revealed that suppression of SHMT1 activity rescues primary root growth in PPSB mutants (see Fig.). Because SHMT1 is mainly expressed in leaves, the authors asked whether Ser metabolism affects nutrient partitioning between roots and shoots. Interestingly, grafting experiments showed that metabolic changes in the shoot are sufficient to rescue root development in double mutants deficient in both the PPSB and GPSB. These results were corroborated by global transcriptomic analysis indicating marked differences in gene expression of PPSB- and GPSB-deficient lines as compared to PPSB-deficient lines in shoots. Mechanistically, the authors postulate that the accumulation of high levels of Gly in GPSB mutants favors Ser biosynthesis in the cytosol and plastids and improves nitrogen, sulfur, and folate metabolism required to support plant growth. It is tempting to speculate that biosynthetic pathways define distinct Ser pools in cells that feedback to regulate specific metabolic pathways.

**Figure.**
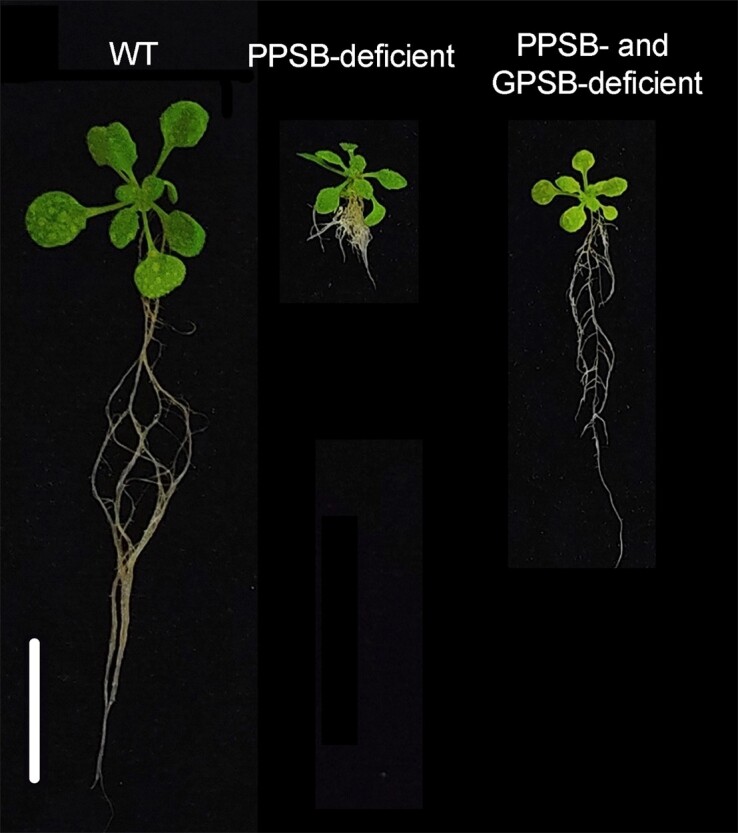
Interplay between Ser biosynthetic pathways affects plant growth. Representative plants of wild-type (WT) and mutants deficient in either the PPSB pathway alone or both PPSB and GPSB pathways, grown under elevated CO_2_. Scale bar = 2 cm. Adapted from Rosa-Téllez et al. (2023), Figure 2B.
